# Epigenetic up-regulation of ribosome biogenesis and more aggressive phenotype triggered by the lack of the histone demethylase JHDM1B in mammary epithelial cells

**DOI:** 10.18632/oncotarget.16181

**Published:** 2017-03-14

**Authors:** Alice Galbiati, Marianna Penzo, Maria Giulia Bacalini, Carmine Onofrillo, Ania Naila Guerrieri, Paolo Garagnani, Claudio Franceschi, Davide Treré, Lorenzo Montanaro

**Affiliations:** ^1^ Department of Experimental, Diagnostic, and Specialty Medicine, Alma Mater Studiorum, University of Bologna, Bologna, Italy

**Keywords:** histone modification, JHDM1B, rDNA, ribosome biogenesis, cancer cells

## Abstract

The alterations of ribosome biogenesis and protein synthesis play a direct role in the development of tumors. The accessibility and transcription of ribosomal genes is controlled at several levels, with their epigenetic regulation being one of the most important. Here we explored the JmjC domain-containing histone demethylase 1B (JHDM1B) function in the epigenetic control of rDNA transcription. Since JHDM1B is a negative regulator of gene transcription, we focused on the effects induced by JHDM1B knock-down (KD). We studied the consequences of stable inducible JHDM1B silencing in cell lines derived from transformed and untransformed mammary epithelial cells. In these cellular models, prolonged JHDM1B downregulation triggered a surge of 45S pre-rRNA transcription and processing, associated with a re-modulation of the H3K36me2 levels at rDNA loci and with changes in DNA methylation of specific CpG sites in rDNA genes. We also found that after JHDM1B KD, cells showed a higher ribosome content: which were engaged in mRNA translation. JHDM1B KD and the consequent stimulation of ribosomes biogenesis conferred more aggressive features to the tested cellular models, which acquired a greater clonogenic, staminal and invasive potential. Taken together, these data indicate that the reduction of JHDM1B leads to a more aggressive cellular phenotype in mammary gland cells, by virtue of its negative regulatory activity on ribosome biogenesis.

## INTRODUCTION

Ribosome biogenesis, the process of ribosome production, is frequently up-regulated in cancer in order to respond to the increased demand of protein synthesis in highly proliferating cells. Indeed, this process is controlled by the same oncoproteins and tumor suppressors that normally control cell cycle progression, cell differentiation, and cell proliferation. Their de-regulation in tumors could be responsible for both a surge in ribosome biogenesis, and the loss of cell cycle control [1].

JmjC domain-containing histone demethylase 1B (JHDM1B, also called FBXL10 or KDM2B) is a conserved and ubiquitously expressed member of the JmjC domain-containing histone demethylase (JHDM) family involved in the demethylation of trimethylated lysine 4 on histone H3 (H3K4me3) and dimethylated lysine 36 on histone H3 (H3K36me2), thereby removing active chromatin marks and inhibiting gene transcription [2–4]. JmjC family members are able to specifically demethylate mono-, di-, and trimethylated lysines on the histone tails [5, 6]. Great importance has recently been attributed to the negative regulatory function of JHDM1B through lysine H3K36me2 demethylation over a group of genes involved in senescence control, mapping at the Ink4a/Arf/Ink4b locus [7, 8]. These genes encode for a series of critical cell cycle regulators: p16^Ink4a^ and p15^Ink4b^ prevent G1/S phase transition through the inhibition of cyclin D binding to cyclin-dependent kinase 4 and 6 (Cdk4/6); p14^Arf^ protein leads to cell cycle arrest or apoptosis, through p53 pathway activation [9, 10]. In this sense, JHDM1B acts as an oncoprotein that prevents proliferative senescence through Ink4a/Arf/Ink4b locus silencing [11]. Accordingly, it has been reported that this specific locus is frequently deleted or mutated in a variety of human primary tumors and that, in leukemia, JHDM1B overexpression drives tumorigenesis by overcoming cellular senescence [12, 13]. On the other hand, a few but important observations involve JHDM1B in tumor suppression. In fact, it is reported to limit cell proliferation through the negative regulation of the transcription of the JUN proto-oncogene and rDNA genes [2, 14, 15]. Ribosomal genes are indeed present in hundreds of copies, but only a subset of the total pool is transcriptionally active at any given time due to their epigenetic inactivation [16]. In particular, the negative regulatory function of JHDM1B in ribosome biogenesis has been linked to H3K4me3 demethylation at the rDNA level. In fact, the demethylase co-localizes with RNA polymerase I (PolI) and the Upstream Binding Factor (UBF) within Nucleolar Organizing Regions (NORs), preferentially binding the transcribed region of ribosomal DNA to repress its transcription [2]. In this sense, considering that low JHDM1B expression levels were found in aggressive brain and breast tumors characterized by an abnormally amplified ribosome biogenesis, we hypothesized that, despite its suppressive activity on proliferative senescence, the downregulation of this histone demethylase might contribute to tumor aggressiveness [2, 15]. To verify the stated hypothesis, we investigated the role of JHDM1B-mediated negative control in ribosome biogenesis and its relevance to cancer, by focusing on the effects of JHDM1B downregulation on ribosome biogenesis and cell behavior in transformed and untransformed cells derived from mammary gland epithelium.

## RESULTS

### JHDM1B downregulation variably affects the global histones marks

**Figure 1:**
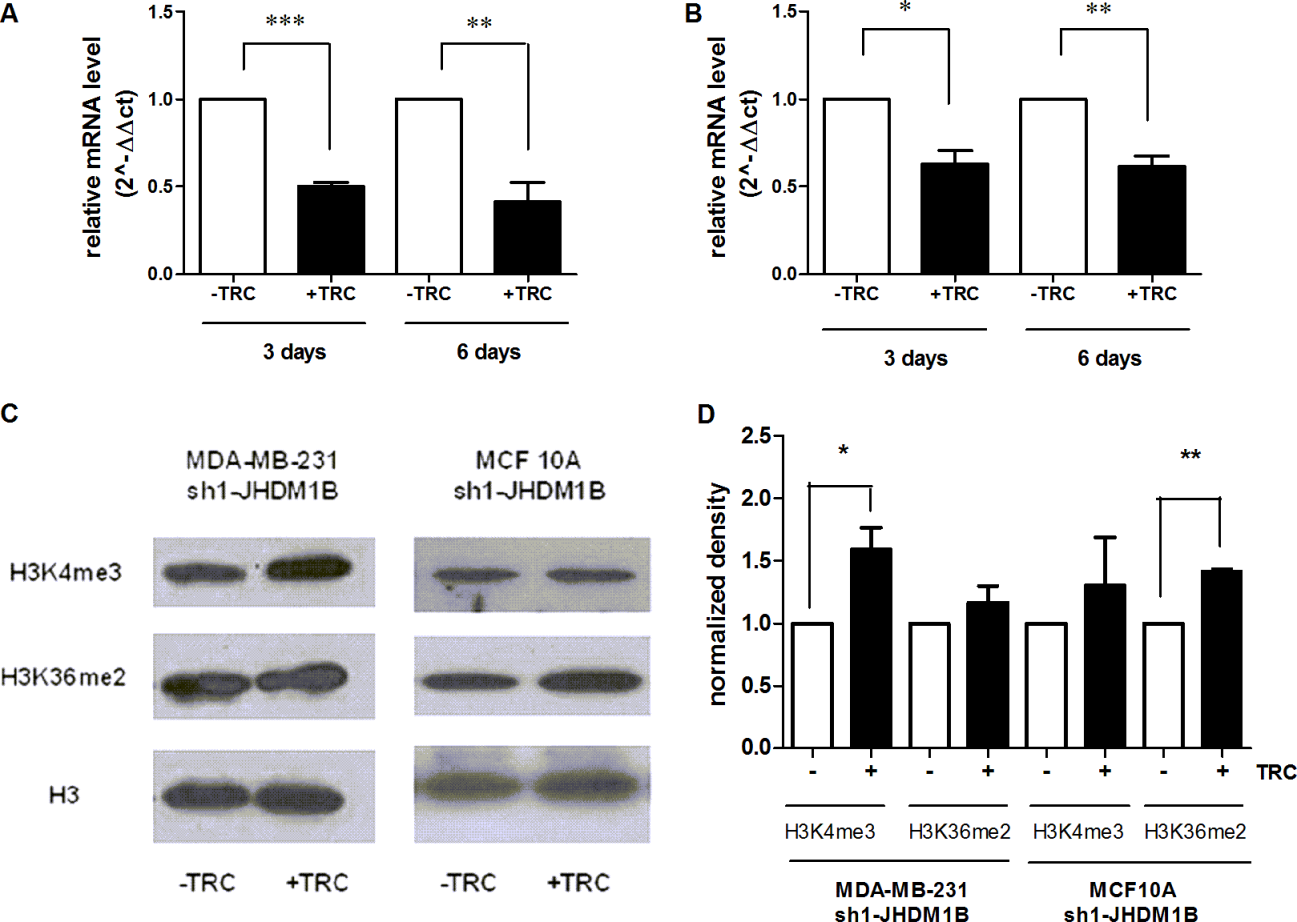
JHDM1B knock-down is associated with histone mark modification (**A** and **B**) JHDM1B mRNA expression measured by real-time RT-PCR after 3 and 6 days of TRC administration in MDA-MB-231 sh1-JHDM1B (A) and MCF10 sh1-JHDM1B (B). Data were analyzed by paired Student's *T-test*: *P < 0.05; **P < 0.01; ***P < 0.001, (error bars, SEM). (**C**) Western blot analysis of the H3K4me3, H3K36me2 and total H3 histone levels in purified histones from MDA-MB-231 sh1-JHDM1B and MCF 10A sh1-JHDM1B cells. (**D**) Densitometry of the gels shown in Figure 1C. The band densities corresponding to the H3K4me3 and H3K36me2 were normalized to those of the total H3 histone and subsequently data were normalized on the relative controls (-TRC). Results were analyzed by paired Student's *T-test* *P < 0.05, (*N* = 3, error bars, SEM).

The purpose of this work was to study the biological effects of long-lasting JHDM1B depletion on the transcription of ribosomal genes, as well as its contribution to the landscape of malignant phenotypes in mammary gland epithelial cells. To study the effects of long-lasting JHDM1B downregulation, we generated TRC-inducible shRNA cellular models starting from MDA-MB-231 and MCF 10A cells [termed hereafter MDA-MB-231 sh(1 or 2)-JHDM1B and MCF 10A sh(1 or 2)-JHDM1B]. After TRC treatment, we observed a drop in JHDM1B mRNA levels starting at 72 h, which was recorded for at least 6 days in both MDA-MB-231 sh1-JHDM1B and MCF 10A sh1-JHDM1B cells (Figure 1A and 1B), thus indicating an early and stable effect of the shRNA expression. JHDM1B KD was associated with some changes in the global levels of the histone methylation set, measured by Western blot (Figure 1C and 1D). In particular, MDA-MB-231-derived cells, after JHDM1B KD, showed increased global levels of histone H3K4me3, while in the MCF 10A the H3K36me2 histone mark increased after JHDM1B KD, supporting a role played by this demethylase in the modification of these specific lysine residues. Nevertheless, in our conditions we did not observe significant changes in the mRNA expression of cyclin-dependent kinase inhibitor 2B (CDKN2B, p15), cyclin-dependent kinase inhibitor 1A genes (CDKN1A, p21), or polycomb silencing complex 1 (PRC1)-related genes, such as the enhancer of zeste homolog 2 (EZH2), and proto-oncogene polycomb ring finger (BMI1) (Supplementary Figure 1), even though these were previously shown to undergo a modulation after acute JHDM1B depletion [15, 17].

### JHDM1B downregulation stimulates 45S pre-rRNA transcription and processing

**Figure 2:**
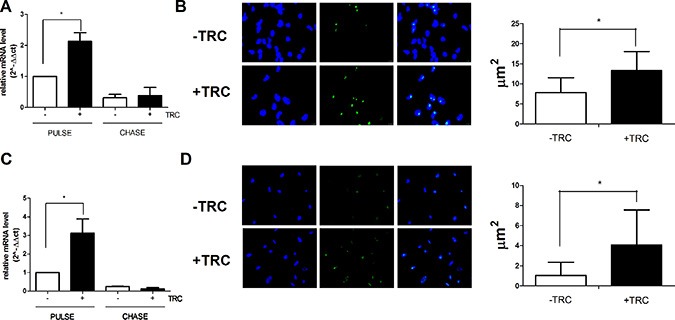
JHDM1B knock-down causes a surge in 45S pre-RNA synthesis and processing (**A**) Evaluation of the neo-produced and processed 45S pre-rRNA in MDA-MB-231 sh1-JHDM1B. Cells were grown in medium supplemented with 5-ethynyl uridine for 1 h (pulse), or additionally grown in the presence of an excess of non-modified uridine for 2 h (chase), in order to evaluate respectively the neo-synthesized and processed 45S pre-rRNA by real time RT-PCR. (**B**) Evaluation of the neo-produced and processed 45S pre-rRNA in MCF 10A sh1-JHDM1B. Cells were grown in medium supplemented with 5-ethynyl uridine for 2 h (pulse), or additionally grown in the presence of an excess of non-modified uridine for 2 h (chase), results were analyzed by paired Student's *T-test*; *P < 0.05; **P < 0.01; ***P < 0.001, (*N* = 6, error bars, SEM). (**C**) 5-fluoro uridine incorporation in nascent RNA in MDA-MB-231 sh1-JHDM1B (left) and average fluorescent nucleolar area (right). (**D**) 5-fluoro uridine incorporation in nascent RNA in MCF 10A sh1-JHDM1B (left) and average fluorescent nucleolar area (right). Dapi staining of nuclei (left blue signal), anti-mouse Alexa-488 (center green signal), merge of the two channels (right). Results were analyzed by unpaired Student's *T-test* *P < 0.05 (error bars, SD).

Pulse-chase experiments aimed at evaluating the consequences of JHDM1B downregulation on rDNA transcription showed that JHDM1B KD affects the transcription of rDNA, by determining a conspicuous increase and a faster processing of neo-synthesized 45S pre-rRNA (Figure 2A, 2C and Supplementary Figure 2). Accordingly, the incorporation of 5-fluoro uridine in nascent RNA was significantly increased after JHDM1B KD (Figure 2B and 2D), thus confirming an increase in rRNA transcription as a result of JHDM1B depletion. To rule out the possibility of off-target effects linked to the use of a single shRNA, we also achieved JHDM1B KD by expressing a different JHDM1B oligo in MDA-MB-231 and MCF 10A cells (MDA-MB-231 sh2-JHDM1B and MCF 10A sh2-JHDM1B); this demonstrated an increased 45S pre-rRNA transcription during pulse-chase experiments also (Supplementary Figure 3).

### JHDM1B downregulation modifies H3K36 methylation at the rDNA level

**Figure 3:**
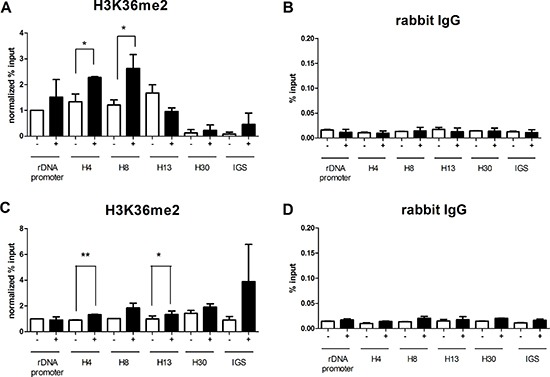
JHDM1B KD causes a modulation of the H3K36me2 mark at the rDNA level (**A**) Chromatin immunoprecipitated with an anti-H3K36me2 in MDA-MB-231 sh1-JHDM1B control (white-filled) and KD cells (black-filled). (**B**) Chromatin immunoprecipitated with normal rabbit IgG in MDA-MB-231 sh1-JHDM1B control (white-filled) and KD cells (black-filled). (**C**) Chromatin immunoprecipitated with an anti-H3K36me2 in MCF10A sh1-JHDM1B control (white-filled) and KD cells (black-filled). (**D**) Chromatin immunoprecipitated with normal rabbit IgG in MCF10A sh1-JHDM1B control (white-filled) and KD cells (black-filled). Quantification obtained by real-time RT-PCR with specific primers for different regions within ribosomal genes: promoter, and three sequences located 4 (H4), 8 (H8), and 13 (H13) kb downstream of the start transcription site, one sequence located in the non transcribed spacer (H30) and one sequence located in the IGS region. Data were expressed as a % of the input without further normalization or normalized over the % of input of rDNA promoter in control cells. The statistical analysis was by paired Student's *T-test* *P < 0.05; **P < 0.01 (*N* = 3, error bars, SEM).

In order to investigate the role of JHDM1B in the modulation of histone markers at specific sites within ribosomal genes, we performed chromatin immunoprecipitation in MDA-MB-231 sh1-JHDM1B KD and MCF10A sh1-JHDM1B KD as well in their relevant controls, using an antibody raised against H3K36me2, the major biochemical target of JHDM1B [3]. The purified chromatin was used in a quantitative PCR analysis in combination with six different pairs of primers mapping at the rDNA promoter, at four sequences located downstream of the start transcription site of the rDNA repeated units 4 (H4), 8 (H8), 13 (H13) and 30 (H30) kb and one mapping at the intergenic spacer region (IGS). The evaluation of the association of different rDNA regions and H3K36me2 revealed a significant increase in the levels of this histone mark in H4 and H8 sequences after JHDM1B depletion in MDA-MB-231 cells (Figure 3A and 3B). No significant changes in H3K36me2 composition were observed in the rDNA promoter region and at the H13, H30 and IGS regions. In MCF10A KD cells a significant increase of H3K36me2 histone mark was observed in H4 and H13 regions, no particular changes in all the other analyzed regions (Figure 3C and 3D). Chromatin immunoprecipitation data obtained against total H3 histone as controls are also reported in (Supplementary Figure 4), showing that a global reorganization of histone H3 association with rDNA occurs after JHDM1B depletion in both cell lines, but with different behaviors.

These results suggest that JHDM1B may control chromatin status at the rDNA level, also acting on H3K36me2.

### JHDM1B affects rDNA methylation

**Figure 4:**
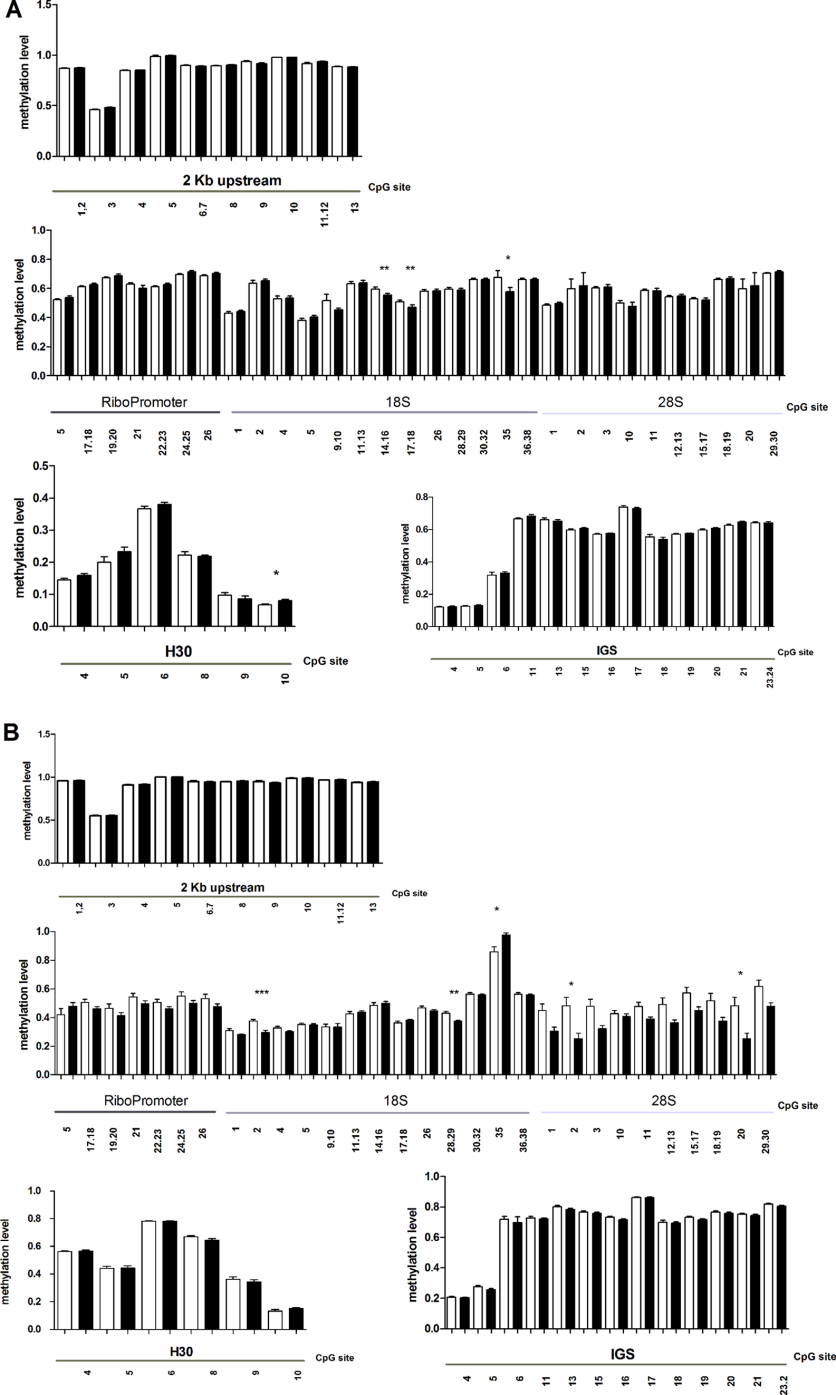
MassARRAY EpiTYPER analysis of KD (black-filled) and control cells (white-filled) (**A** and **B**) JHDM1B knock-down changes the methylation status of ribosomal genes: (A) MDA-MB-231 sh1-JHDM1B; (B) MCF 10A sh1-JHDM1B. Statistical analysis was by paired Student's *T-test* *P < 0.05; **P < 0.01; ***P < 0.001, (*N* = 5, error bars, SEM).

Since JHDM1B histone demethylase can cause induce modifications in chromating status, it is conceivable that the methylation pattern of rDNA may also change in JHDM1B KD cells. We used the EpiTYPER assay to quantitatively measure the methylation level of single CpG sites and small groups of adjacent CpGs (CpG units) in rDNA. In particular, we analyzed six target regions in ribosomal genes (Figure 4): a region 2 kb upstream of the upstream promoter of the ribosomal genes (2 kb upstream), a region including both the upstream and the core promoters of ribosomal genes (RiboPromoter), the 5′ of 18S sequence (18S), the 5′ of 28S sequence (28S), and two regions overlapping with the H30 and the IGS sequences analyzed in chromatin immunoprecipitation experiments. Some of these regions partially overlap those previously analyzed in other studies [18]. After data cleaning we were able to measure the methylation levels of 10 CpG units (containing 13 CpGs), 8 CpG units (containing 13 CpGs), 14 CpG units (containing 26 CpGs), 10 CpG units (containing 15 CpGs), 6 CpG units (containing 6 CpGs) and 13 CpG units (14 CpGs) in 2 kb upstream, RiboPromoter, 18S, 28S, H30 and IGS target regions, respectively. In RiboPromoter amplicon, 7 CpGs were localized in the upstream control element (UCE) region, while the remaining 6 were in the core promoter. This analysis revealed that the methylation status of many CpG sites in rDNA changes after JHDM1B KD in MDA-MB-231 sh1-JHDM1B and MCF 10A sh1-JHDM1B cells. In particular, in MDA-MB-231 sh1-JHDM1B KD cells three CpG units were demethylated in the 18S amplicon of MDA-MB-231 sh1-JHDM1B KD (18S_14.16; 18S_17.18, and 18S_35) and one was hypermethylated in the H30 amplicon (H30_10) (Figure 4A). In MCF 10A sh1-JHDM1B KD two CpG units of the 18S were found to be demethylated (18S_2, 18S_28.29), and one unit of the same amplicon was hypermethylated (18S 35) after JHDM1B downregulation. The whole 28S amplicon appeared to be particularly affected by JHDM1B KD with many modified CpGs, including two sites (28S_2 and 28S_20), which were subjected to a significant demethylation (Figure 4B). Overall these results suggest that JHDM1B depletion induces a relaxation of rDNA chromatin at specific sites in the transcribed region thus allowing the access to DNA modifier enzymes. In the RiboPromoter, in the 2 kb upstream region and in the IGS region we were not able to find significantly different methylation patterns between control and silenced cells, suggesting that DNA methylation changes are locus-specific. The differences in methylation recorded at specific sites between the two cell lines may be ascribed to a different cell line-specific epigenetic asset.

### Polysome profile analysis revealed an increased number of ribosomes involved in protein translation after JHDM1B KD

**Figure 5:**
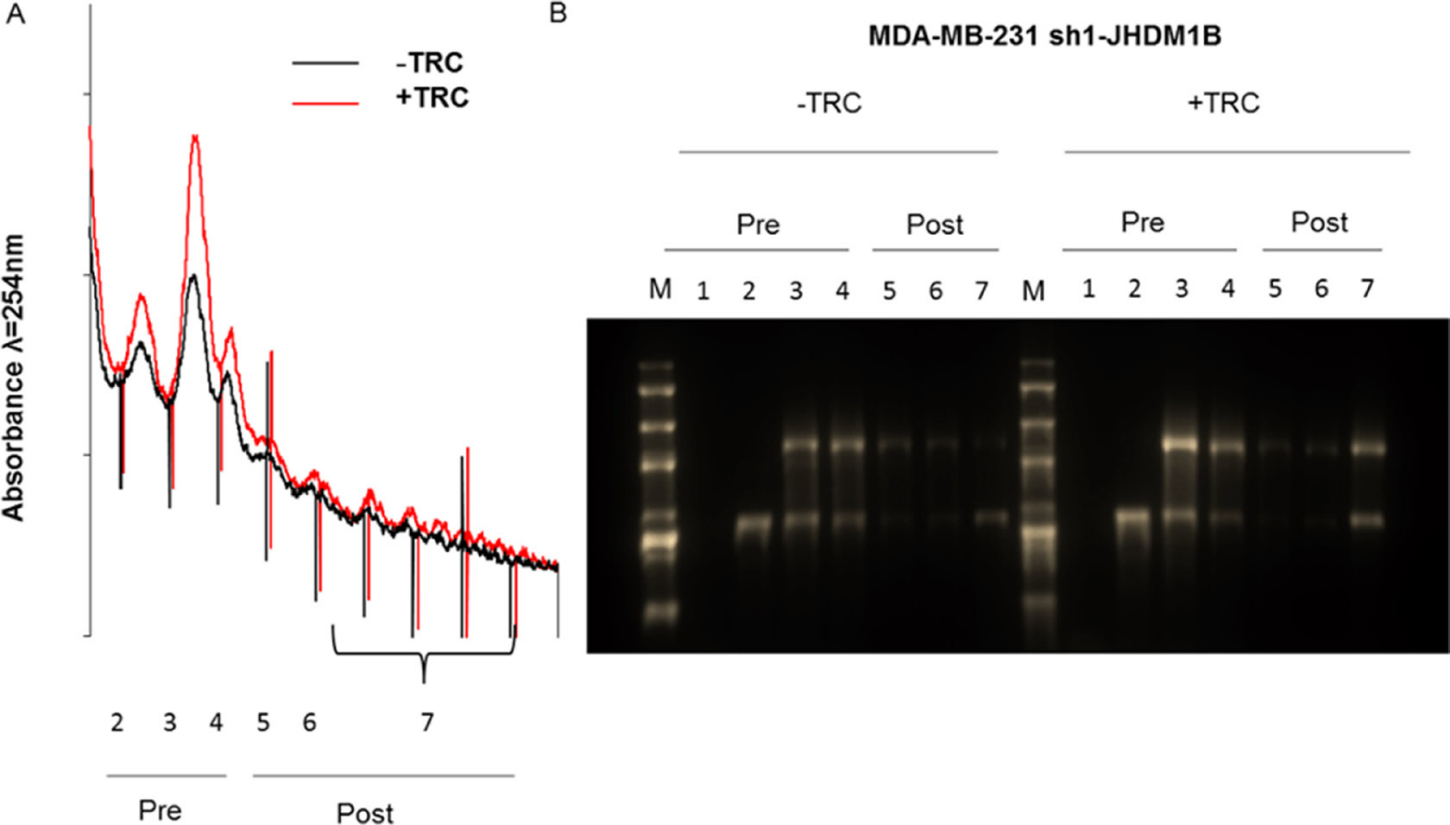
Polysome profile analysis of MDA-MB-231 sh1-JHDM1B cells (**A**) Polysome profiles of control (black line) and KD cells (red line) obtained by measuring the absorbance at 254 nm in a continuous 15%–50% sucrose gradient. Numbers correspond to the sedimentation coefficients as follows: pre-polysomial 1) < 40 S; 2) 40 S; 3) 60 S; 4) 80 S, and post-polysomial > 80 5) disomes; 6) trisomes; and 7) the remaining polysome fractions. (**B**) Denaturing formaldehyde 1% agarose gel loaded with the total RNA extracted from each fraction obtained from the polysomal profile (M: 10000 bp RNA marker). Polysome profiles and gel are representative images of 3 independent experiments.

In order to understand if the boosted transcription of 45S pre-rRNA precursor was accompanied by an increase in ribosome production, we performed polysomal profile analysis via density fractioning of cellular lysates extracted from the same number of control and JHDM1B KD MDA-MB-231 cells. This analysis revealed an increase in both the pre-polysomal and polysomal fractions absorbance in JHDM1B KD cells (Figure 5A); in addition, an enrichment of 18S and 28S rRNAs has been found in all the gradient fractions of the silenced MDA-MB-231 sh1-JHDM1B cells, particularly evident in the pooled polysomal fractions (Figure 5B), thus indicating that a higher number of ribosomes were involved in protein translation after JHDM1B reduction. Consistent results were observed when analyzing the amount of total and ribosomal RNA obtained from the same number of control and JHDM1B KD MCF10A cells (Supplementary Figure 5).

### JHDM1B downregulation triggers an aggressive behavior of mammary epithelial cells

**Figure 6:**
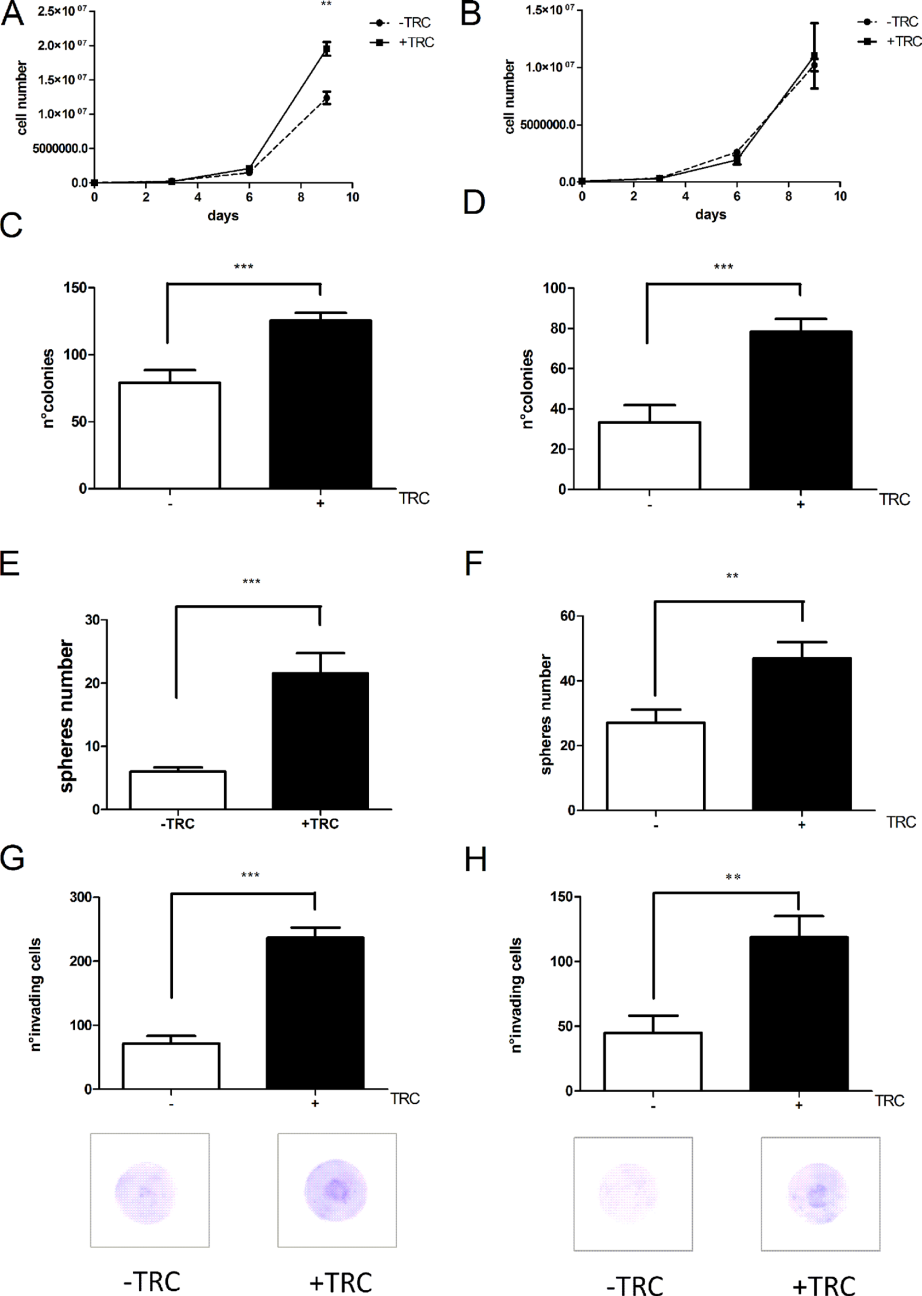
JHDM1B silencing triggers a more aggressive phenotype in the tested cell lines (**A**) Growth curve profiles of control MDA-MB-231 sh1-JHDM1B cells (dotted line) and KD cells treated with TRC (solid line). (**B**) Growth curve profiles of control MCF 10A sh1-JHDM1B cells (dotted line) and KD cells (solid line) (*N* = 3, error bars, SEM). (**C** and **D**) Colony numbers obtained from (C) MDA-MB-231 sh1-JHDM1B and (D) MCF 10 A sh1-JHDM1B (*N* = 3, error bars, SEM). (**E** and **F**) Number of mammospheres produced by MDA-MB-231 sh1-JHDM1B (E) and MCF 10A sh1-JHDM1B (F). (**G** and **H)** Matrigel invasion assay of MDA-MB-231 sh1-JHDM1B (G) and MCF 10A sh1-JHDM1B (H). All graphs in the figure were obtained averaging at least three independent experiments (and no internal normalization between different experiments was performed), *p values* were obtained by unpaired Student's *T-test* *P < 0.05; **P < 0.01; ***P < 0.001 (error bars, SEM). The images below provide a representative picture of the matrigel-coated filters after 16 h of invasion. Photographs of 5 different areas were acquired for each filter, at 10× magnification, and used for cell counts. (*N* = 6, error bars, SEM).

The inducible system we developed allows the analysis of prolonged JHDM1B depletion on different aspects of tumor cell behavior. We then explored various functional aspects such as growth and invasion capacity, ability to form colonies from individual cells, and mammosphere formation, in control and JHDM1B-depleted MDA-MB-231 and MCF 10A cells. JHDM1B KD sustained over time caused an increase in MDA-MB-231 sh1-JHDM1B cellular proliferation (Figure 6A). No effect on the growth behavior of MCF 10A sh1-JHDM1B was observed (Figure 6B). JHDM1B KD led to a significantly enhanced colony-forming ability in both cell lines (Figure 6C and 6D). MDA-MB-231 and MCF 10A sh1-JHDM1B cells, as their relative controls, were still able to form mammospheres (MS) when grown on ultra-low attachment plates. MS can be formed from a niche of cells, with a staminal potential, able to grow as colonies in suspension in a syncytial state; in this condition the colony assumes a spherical shape [19, 20]. Both MDA-MB-231 and MCF 10A sh1-JHDM1B treated with TRC form a higher number of MS compared to control cells, indicating a greater staminal potential (Figure 6E and 6F). Lastly, the invasion assays revealed a significant increase in the number of invading cells after JHDM1B KD in sh1-JHDM1B models (Figure 6G and 6H). To rule out the possibility of off-target effects, we conducted invasion assays in MDA-MB-231 sh2-JHDM1B and MCF 10A sh2-JHDM1B, obtaining similar results (Supplementary Figure 6). Over all, these experiments indicate that the reduction of JHDM1B levels triggers an aggressive behavior of mammary gland epithelial cells, which is typical of cancer cells. Interestingly, MCF 10A sh1-JHDM1B, an untransformed cell line usually unable to form colonies and invade, acquired these skills after JHDM1B KD.

**Figure 7:**
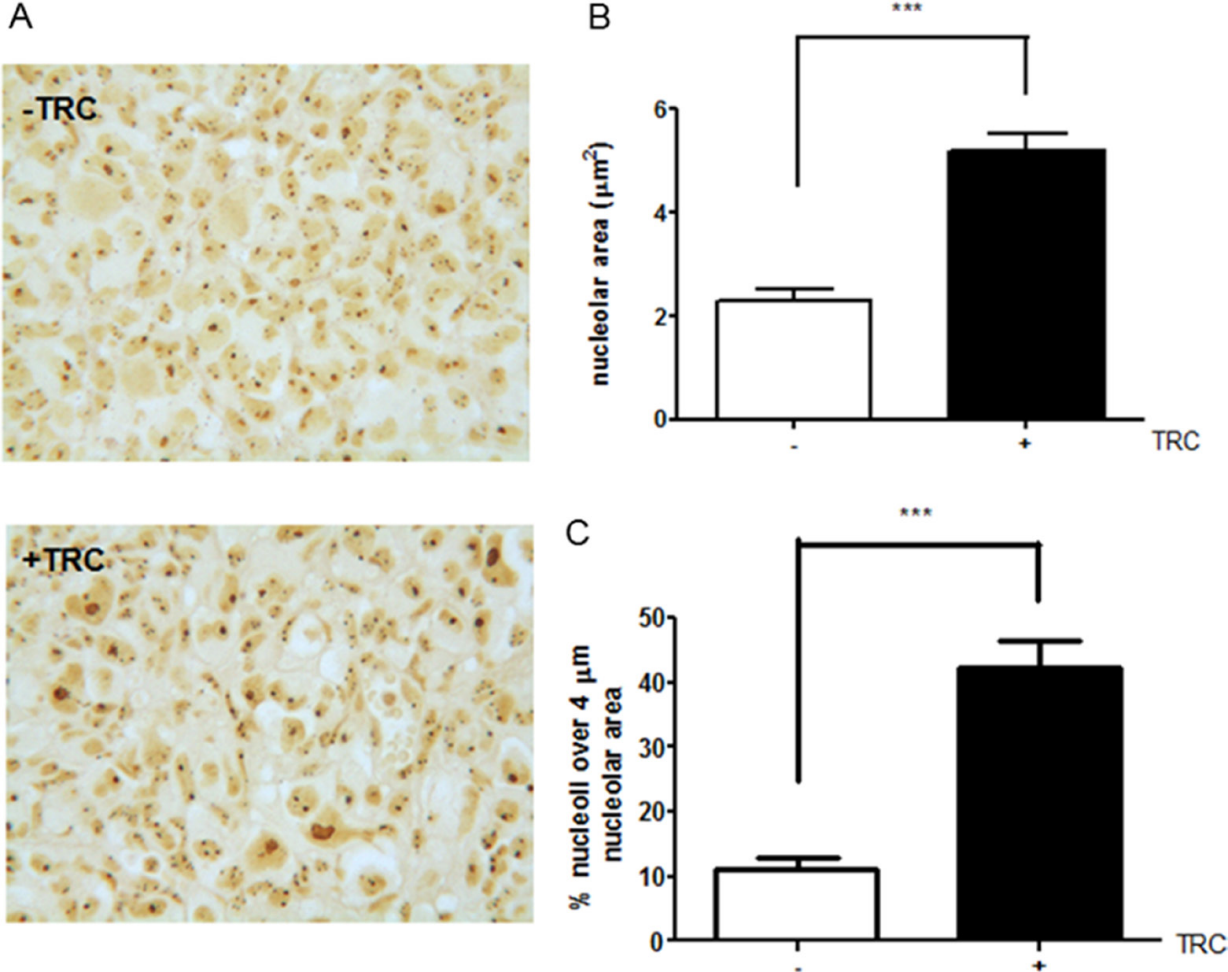
JHDM1B knock-down in MDA-MB-231 sh1-JHDM1B xenograft (**A**) Representative images of AgNOR staining in MDA-MB-231 sh1-JHDM1B control tumor (upper panel) and TRC-treated mice (lower panel). Images show the presence of large nucleoli in JHDM1B KD xenograft. (B) Average nucleolar area measured in peripheral regions of 10 different tumors for each treatment. (**C**) Average percentage of large nucleoli (greater than 4 μm) in the total counted nucleoli. *p values* were obtained using the unpaired Student's *T-test* ***P < 0.001 (error bars, SD).

Since JHDM1B-depleted cells showed a more aggressive behavior, a MDA-MB-231 sh1-JHDM1B tetracycline-inducible shRNA model was used to generate tumors in nude Balb/c mice by injecting 2 × 10^6^ cells into each of the animals’ flanks (Supplementary Data). After 9 weeks the mice were euthanized and the isolated tumors were measured. The obtained results indicated that JHDM1B KD triggered an incremental (although not statistically significant) trend in tumor growth (Supplementary Figure 7), while the silver nitrate staining of tumor sections (a technique widely used for selective nucleolar staining) revealed a different morphological conformation of the nucleoli after JHDM1B KD (Figure 7A). Tumors from TRC-treated mice showed a significant increase in the average nucleolar area compared to the untreated mice (Figure 7B), with a higher percentage of very large nucleoli (> 4 μm^2^) (Figure 7C).

## DISCUSSION

In this study we observed that the TRC-driven JHDM1B silencing in our cellular models caused a surge in 45S pre-rRNA transcription, the rate-limiting step of ribosome biogenesis. The histone marks known to be the targets of JHDM1B were consequently modulated at different specific lysine residues looking to the global histone H3 methylation. In particular, the increased levels of H3K4me3 in MDA-MB-231 sh1-JHDM1B and higher level of H3K36me2 in MCF 10A sh1-JHDM1B were found after JHDM1B KD. On this concer, it should be considered that changes in the total levels of histone modifications after JHDM1B KD are not necessarily to be expected. Indeed, in the absence of global changes, it is still possible that focused lysine residue modifications occur at specific loci. Previous studies on Hela cells overexpressing JHDM1B mutated in its catalytic domain, demonstrated a decrease in the H3K4me3 level without affecting the global H3K36me2 mark [2] compared to the control cells expressing the wild type protein. In mouse embryo fibroblasts (MEF), no changes in the global levels of histone modification were found after JHDM1B overexpression, while in experiments of chromatin immunoprecipitation, a different H3K4me3 and H3K36me2 composition was observed at specific loci [4]. To study histone mark changes at the rDNA level, we used chromatin immunoprecipitation and demonstrated for the first time that JHDM1B-downregulated cells increased the H3K36me2 level at the 4 kb and 8kb transcribed regions of rDNA after KD, thereby proving that JHDM1B acts as a negative regulator of ribosome biogenesis not only by targeting the known H3K4me3 residue, but also by acting on its major substrate H3K36me2. This finding is strengthened by the recent observation that JHDM1A, a histone demethylase close to JHDM1B, acts on the same lysine residue in the negative regulation of rDNA transcription in response to glucose and serum starvation [21].

In addition, low levels of JHDM1B cause changes in rDNA methylation patterns, mainly resulting in a decreased level of methylation in the 18S and 28S CpG islands, which falls in the rDNA regions known to be directly bound by JHDM1B [2]. The obtained general decrease in rDNA methylation is in line with a reduced chromatin condensation which is consequent to the lack of JHDM1B repressive function at the rDNA level. This observation is consistent with the increased rRNA transcription observed in JHDM1B KD. In the RiboPromoter region we were not able to find a significantly different methylation pattern between control and silenced cells. These results are in line with the available data that identify the 18S and 28S regions as primary binding sites for JHDM1B in rDNA, while a very low interaction is found between the demethylase and the promoter region [2].

JHDM1B KD did not represent a harmonic stimulus to the ribosome biogenesis, since in silenced cells the first effect is a boost in 45S pre-rRNA production which is not expected to be paralleled by a concerted stimulation of all those factors which are important for a proper ribosome biogenesis. Despite this, we demonstrated that a higher number of ribosomes were present in cells with reduced JHDM1B levels and were involved in the protein synthesis process.

We also proved that the enhanced ribosome biogenesis conferred a growth advantage to MDA-MB-231 cells, while this effect was not observe in MCF10A. Since these two cell lines display a different p53 status (wild type for MCF10A and mutated for MDA-MB-231-[22], this observation is in line with our previous study showing that the effects of JHDM1B depletion on cellular proliferation are dependent on the status of p53 [15]. In addition, both tested cell lines acquired a greater clonogenic, staminal and invasive potential. Taken together, these data support the role of JHDM1B as a tumor suppressor, since it is a negative regulator of ribosome biogenesis and cell proliferation. Apparently, the data obtained in our laboratory are in contrast with those reported by Kottakis *et al*., where JHDM1B silencing through a stable, and not inducible, lentiviral system in the same cell lines led to cell death by the activation of the Ink4a/Arf/Ink4b locus and PRC1 [17]. It is worthy of note that, in this study, a greater efficiency in gene silencing was obtained compared to that achieved with our inducible system, suggesting that the differences in the experimental approaches employed may be responsible for the different results observed

On the other hand, our data are consistent with those obtained after transient KD experiments in the same cellular models and with those that correlate JHDM1B mRNA levels in primary breast cancer specimens and patient clinical outcomes [15]. In particular, a worse prognosis was observed in cases in which the levels of the histone demethylase expression were below the median of the distribution, a situation comparable to the partial KD that we obtained in the present study.

In conclusion, the role of JHDM1B as an oncogene or tumor suppressor is still unclear and the subject of a widespread scientific discussion, but the data provided in this study support its role against breast cancer development and progression.

## MATERIALS AND METHODS

### Cell culture and inducible shRNA interference

MDA-MB-231 and MCF 10A cell lines were obtained from the American Type Culture Collection. During the study, cell line identity was verified by DNA fingerprinting and/or by day-by-day morphology checking. Cell lines expressing tetracycline inducible anti-JHDM1B shRNA, MDA-MB-231 sh(1 or 2)-JHDM1B, and MCF 10A sh(1 or 2)-JHDM1B were generated by the BLOCK-IT inducible H1 Lentiviral RNAi System following the manufacturer's instructions (Life Technologies). MDA-MB-231 sh(1 or 2)-JHDM1B were cultured in DMEM supplemented with 10% Fetal Bovine Serum (FBS), 2 mM L-Glutamine, 100 U/ml Penicillin and 1 mg/ml Streptomycin, 12 μg/ml Blasticidin, and 100 μg/ml Zeocin ( Sigma-Aldrich). MCF 10A sh(1 or 2)-JHDM1B were cultured in DMEM 1 g/L glucose supplemented with 250 U/L of insulin, 0,5 μg/ml of hydrocortisone, 10 ng/ml of epidermal growth factor, 20% (FBS), 2 mM L-Glutamine, 100 U/ml Penicillin, and 1 mg/ml Streptomycin, 8 μg/ml of Blasticidin and 100 μg/ml Zeocin (Sigma-Aldrich). The expression of anti-JHDM1B shRNAs was obtained by the daily administration of Tetracycline (TRC) 1 μg/ml for sh1-JHDM1B cell lines, and 16 μg/ml for sh2-JHDM1B. All experiments were carried out after 6 days of pre-induction of JHDM1B KD with TRC. To rule out the possibility that TRC treatment, rather than JHDM1B KD, affected cell proliferation, we investigated the effect of TRC administration on cell proliferation (Supplementary Figure 7A and 7B), and 5-fluoro uridine incorporation (Supplementary Figure 8C and 8D) in the parental cell lines MDA-MB-231 and MCF 10A. The double stranded DNA sequences used for the expression of the anti-JHDM1B shRNA were: sh1-JHDM1B forward: CACCGCAAACAGAGTGACATCTTTCCGAAGAAA GA; sh1-JHDM1B reverse: AAAAGCAAACAGAGTGA CATCTTTCTTCGGAAAGAT; sh2-JHDM1B forward: CACCGCATGAAGCAGAGCTGCATCACGAATGAT GC; sh2-JHDM1B reverse: AAAAGCATGAAGCAGAG CTGCATCATTCGTGATGC. All experiments were performed after the validation of the KD efficiency by real-time RT-PCR. Detailed protocols for cell proliferation, invasion, mammosphere production and colony formation assay were presented in the Supplementary Information (SI) section.

### RNA extraction and real-time RT-PCR

RNAs were extracted using Tri-Reagent (Ambion), following the manufacturer's specifications. Real-time RT-PCR was performed as previously described [23]. Briefly, after RNA extraction and cDNA synthesis, a semi-quantitative Taqman approach (TaqMan Universal PCR master mix, Applied Biosystems) was used to evaluate the expression of JHDM1B, while a Sybr Green approach (Power SYBR green PCR master mix, Applied Biosystems) was used for the evaluation of β-actin as an endogenous control and 45S pre-rRNA. Forward and reverse PCR primer sequences for Sybr green analysis of each mRNA were as follows: actin forward: ATCGTCCACCGCAAATGCTTCTA; actin reverse: AGCCATGCCAATCTCATCTTGTT; 45S pre-rRNA forward: GAACGGTGGTGTGTCGTTC; 45S pre-rRNA reverse: GCGTCTCGTCTCGTCTCACT.

### Histones extraction and western blot

Cells were harvested and re-suspended in a TEB buffer (0.5% Triton-X100, 2 mM phenylmethyl sulfonylfluoride, 0,02% w/v NaN3 in phosphate buffer) at a density of 1 × 10^7^ cells/ml. Cells were then lysed on ice for 10 min and centrifuged at 10000 rpm for 1 min at 4°C; then the supernatant was removed. Cell pellets were suspended in 3 volumes of 10% glycerol, 0.5 N HCl, and incubated on ice for 30 min. Samples were centrifuged at 12000 rpm for 5 min, and supernatants were placed into new tubes containing 8 volumes of acetone for over-night precipitation at −20°C. Samples were centrifuged at 12000 rpm 20 min; then supernatants were removed and the dried pellets were resuspended in distilled water. Histones were quantified with Bradford staining (BioRad) following the standard protocol. 5 μg of total histones were loaded on 10% acrylamide gel to be evaluated by Western blotting histone H3 (Abcam, ab1791), H3K4me3 (Abcam, ab8580), and H3K36me2 (Abcam, ab9049).

### 45S pre-rRNA transcription and processing evaluation

The level of newly synthesized 45S pre-rRNA was evaluated using the Click-iT Nascent RNA Capture Kit following the manufacturer's specifications (Life Technologies). 3 × 10^5^ cells were seeded in a 35 mm dish the day before the experiments. Cells were treated with 0.1 mM 5-ethynil uridine in culture medium for 1 h or 2 h (pulse), respectively, for MDA-MB-231 sh(1 or 2)-JHDM1B and MCF 10A sh(1 or 2)-JHDM1B. For the evaluation of 45S pre-rRNA processing, cells were cultured for 2 additional hours in the presence of an unmodified uridine excess at a concentration of 0.2 mM (chase).

### Immunofluorescent detection of rRNA synthesis

Immunodetection of the nascent rRNA was performed by incorporating 5-fluoro uridine, according to a method described elsewhere [24]. Samples were observed under a Leica DMI4000B inverted fluorescence microscope (Leica Microsystems, Milan, Italy) and analyzed using Image-Pro Analyzer Software.

### Chromatin immunoprecipitations of H3K36me2

Chromatin immunoprecipitations were performed on MDA-MB-231 sh1-JHDM1B and MCF10A sh1-JHDM1B cells using the EZ-Chip Kit following the manufacturer's instructions (Merk-Millipore). In particular, immunoprecipitations were performed by incubating the precleared chromatin, diluted 1:15, overnight at 4°C with 10 μg of specific primary antibodies [rabbit anti-H3K36me2 (Abcam, ab9049), rabbit anti-H3 (Abcam, ab1791) and normal rabbit IgG (Millipore, 12-370)], while 10 μl (1%) of each sample were set aside as “input”. The immunoprecipitated DNA was extracted with a QIAquick PCR purification kit (Qiagen) and used for quantitative RT-PCR analysis. Primer sequences used for H4, H8, and H13 regions have been published elsewhere [25]. Primer sequences for other rDNA regions were: rDNA promoter forward: GGTATATCTTTCGCTCCGAG; rDNA promoter reverse: AGCGACAGGTCGCCAGAGGA. H30 forward: ACTGGCGAGTTGATTTCTGG; H30 reverse: CGAGACAGTCGAGGGAGAAG; IGS forward: CACTACCCACGTCCCTTCAC; IGS reverse: GAGAGAAGACGGAGGCACAC.

### EpiTYPER assay for the quantitative DNA methylation analysis

Quantitative methylation analysis of rDNA locus was performed using the EpiTYPER assay (Sequenom). Briefly, 1000 ng of DNA were bisulfite-converted using the EZ-96 DNA Methylation Kit (Zymo Research Corporation) as previously described [26]. 10 ng of bisulfite-treated DNA were PCR-amplified using already validated primer pairs, three of which were previously analyzed using the same technique [27]: i) 2 kb upstream, from position -2274 to position -2055 (with respect to the transcription start site), forward primer AGGAAGAGA GGGGAGTTGGAGATTAGTTTGAGTAA, reverse primer CAGTAATACGACTCACTATAGGGAGAAGGC TTAAAACAAAATTTCACTCTTATTTCCAC; ii) RiboPromoter, from position −186 to position +48, including both the upstream and core promoters of the ribosomal gene [27]; iii) 18S, from position +2946 to position +3432, encompassing the 5′-sequence of the 18S region [27]; iv) 28S, from position +7297 to position +7579, encompassing the 5′-sequence of the 28S region [27]; v) the H30, forward primer AGGAAGAGAGAGGA TTTTGGGTAGGAAAGTTTTT, reverse primer CAGT AATACGACTCACTATAGGGAGAAGGCTCCCAAATT TAATCTCCCTACAAAATA; vi) the IGS, forward primer AGGAAGAGAGAGGGGGGTTTTATTGTTTTTTGA, reverse primer CAGTAATACGACTCACTATAGGGAG AAGGCTAAAAAAAACCTCACAACTACAAACC.

For each target region, the assay returns the methylation level (expressed as a continuous number ranging from 0 to 1) of either single CpG sites or small groups of adjacent CpG sites. The quality control of EpiTYPER results was performed to remove CpG sites/units with missing values in more than 20% of the samples, as well as samples with missing values in more than 20% of CpG sites/units.

### Polysome profile analysis

A separation based on the sedimentation coefficients of active translation machinery was performed in 15% to 50% continuous sucrose gradient in LSB 1X + Cycloheximide (CHX) [20 mM Trizma Base pH 7.5, 10 mM NaCl, 3 mM MgCl_2_, 100 μg/ml CHX and 0.04 U/μl RiboLock RNAse inhibitor (Thermo Scientific)]. 5 × 10^6^ MDA-MB-231 sh1-JHDM1B KD and control cells were seeded in a 150 mm cell culture dish. After 24 hours, cells were treated with CHX (100 μg/ml) for 20 min at 37°C, 5% CO_2_, washed twice with PBS + CHX (100 μg/ml), and harvested by scraping. The entire procedure was performed on ice and in the presence of CHX for RNA-ribosome complex maintenance. Cell pellets were re-suspended in 150 μl of LSB 1X supplemented with 0.3% Triton N-101, 50 mM sucrose, 100 μg/ml CHX and 0.04 U/μl RiboLock RNase inhibitor and protease inhibitor cocktail (Roche), and incubated for 10 min on ice. Total proteins were quantified by Bradford assay before loading lysates on gradients. Lysates were cleared by centrifugation at 14000 g for 10 min at 4°C. Ribosomes were then separated on the continuous sucrose gradient by ultracentrifugation at 160000 g for 2 h at 4°C. Polysome profile was monitored at 254 nm (0.2 OD sensitivity) and fractionated (at 10×, 10% TRIS-Pump power) using an ISCO gradient fractionator system interfaced to an UA-6 absorbance detector (Teledyne Isco, Lincoln, NE, USA). Collected data were digitally converted by using Minilab 100 (Measuring Computing, Norton, MA, USA) and TracedDaq software (Measuring Computing, Norton, MA, USA), by acquiring data in differential mode at +/− 4 V and 4Hz.

RNA extraction by polysomal fractions and electrophoresis were discussed in the Supplementary Information section (SI).

### Selective nucleolar staining

Five-micron sections were processed and silver-stained to visualize nucleolar organizer regions and argyrophilic proteins according to the guidelines of the “International Committee on AgNOR Quantitation” [28]. Silver-stained sections were examined with a Leitz Diaplan light microscope (Wetzlar Germany) equipped with a video camera (JVC, 3CCD, KY-F55B, Jokohama, Japan), and analyzed using Image-Pro Analyzer Software.

### Statistical analysis

Appropriate statistical analyses were performed as indicated in the figure legends, using the paired or unpaired Student's *T-test*. Data analyses were performed by GraphPad Prism 5.0 and Microsoft Excel.

## SUPPLEMENTARY MATERIALS FIGURES


