# Supplementary material for: Epigenetic up-regulation of ribosome biogenesis and more aggressive phenotype triggered by the lack of the histone demethylase JHDM1B in mammary epithelial cells
Source: Oncotarget. 2017 Mar 14;8(23):37091–103. doi: 10.18632/oncotarget.16181 (PMC5514893; doi:10.18632/oncotarget.16181)
Supplement: Supplementary file 1 [file oncotarget-08-37091-s001.pdf]

Epigenetic up-regulation of ribosome biogenesis and more aggressive phenotype triggered by the lack of the histone demethylase JHDM1B in mammary epithelial cells

Supplementary Materials

SUPPLEMENTARY DATA

MATERIALS AND METHODS

Cellular growth determination

5×10^4 cells pre-treated with TRC or left untreated were seeded in six-well plates and cultured for 10 days in control or TRC conditions. Every 3 days the cells were harvested, counted (after Trypan blue staining to exclude dead cells from the count) in a Burker Haemocytometer, and seeded again in new plates in a 1:4 (MCF 10A derived cells) or 1:5 (MDA-MB-231 derived cells) ratio. Between different points of the growth curves the doubling times (DTs) were calculated using the following formula: DT = [h × ln (a / b)] / ln (2), where h is the growth time in hours, a is the number of seeded cells, and b is the number of cells at the end of the growth time. The DTs were then used to indirectly assess the real cell number by applying the following formula: cell number = $2^{(h/DT)}$.

Cell invasion assay

Invasion assays were performed in blind well chambers (Neuroprobe Inc.) according to the manufacturer's instructions, using 13mm-diameter polycarbonate filters (Neuroprobe Inc.) with pore size 8 µm. 5×10^4 cells, pre-treated with TRC or left untreated, were seeded in the upper compartment in low FBS cell culture medium, [1% and 2% respectively for MDA-MB-231 sh(1 or 2)-JHDM1B and MCF 10A sh(1 or 2)-JHDM1B], while 10% and 20% FBS (respectively) in cell culture medium were placed in the lower compartment. After a 24 h incubation at 37°C, 5% CO_2, filters were collected and washed with water, while cells were fixed in absolute ethanol for 1 min. Lastly, cells were stained with Giemsa stain (1:10 in water) at RT for 10 min and filters were washed again twice with water. The non-invading cells were scraped off with a cotton swab. Cells were visualized with a Leitz Diaplan light microscope (Wetzlar Germany) equipped with a video camera (JVC, 3CCD, KY-F55B, Jokohama, Japan) at 10× of megnification; 5 random fields for each filter were photographed and counted.

Clonogenic assays

In a single 6-well plate, 150 cells or 500 cells, for MDA-MB-231 sh1-JHDM1B and MCF 10A sh1-JHDM1B respectively, were seeded and treated daily with TRC or left untreated. The colony number was evaluated 10–12 days later, after overnight fixation in 4% formalin at 4°C and staining with a 0.5% crystal violet solution in 25% methanol for 30 min. Cells were then washed 3 times in PBS and counted.

Generation of mammospheres

1.2×10^4 cells were seeded in ultra-low attachment 6-well plates and cultured in Mammary Epithelial Cell Growth Medium (MEGM, Bullet Kit, Lonza). Spheres started forming after 4–6 days and MS were counted between days 7 and 8 under an inverted microscope at 10× magnification.

RNA extraction by polysomal fractions and electrophoresis

RNA from polysomal or pre-polysomal fractions was loaded in formamide loading dye (50% formamide, 330 mM EDTA pH 8.0, 0.25 mg/ml bromophenol blue/xylene cyanol and 0.0025% ethidium bromide) and electrophoretic separation was performed in 1% agarose gel with 6% formaldehyde in MOPS 1X (20 mM MOPS pH 7.0, 2 mM $NaCOOCH_3$, 1 mM EDTA pH 8.0). The procedure was performed on ice at constant 100 mV for 1 h.

MDA-MB-231 sh1-JHDM1B xenografts

2×10^6 MDA-MB-231 sh1-JHDM1B cells, pretreated with TRC or left untreated for 6 days, were suspended in 100 µl of PBS and injected subcutaneously into both flanks of anesthetized 5-week-old female Balb/COlaHsd-Foxn1nu mice (Harlan Laboratories Inc.). Five mice for each of the 2 groups (pre-treated with TRC or left untreated) were injected, for a total of 10 xenografts

in control conditions and 10 xenografts in JHDM1B KD condition. Mice of the two groups were watered with 3% sucrose in water or 3% sucrose supplemented with TRC 1.5 mg/ml, respectively, starting 3 days before the xenograft. Mice were euthanized nine weeks later, and the tumor masses were excised from the flanks. Tumor diameters were evaluated using a caliber. Some of the tissues were immediately frozen in liquid nitrogen for subsequent RNA extraction by the Tri-Reagent (Ambion), following the manufacturer's specifications. The remaining tissues were embedded in 4% paraffin and used to generate five-micron sections, later processed to perform the selective nucleolar staining.

All of the animal work was approved by Bologna University's Institutional Animal Care and Use Committee in accordance with national guidelines and standards.

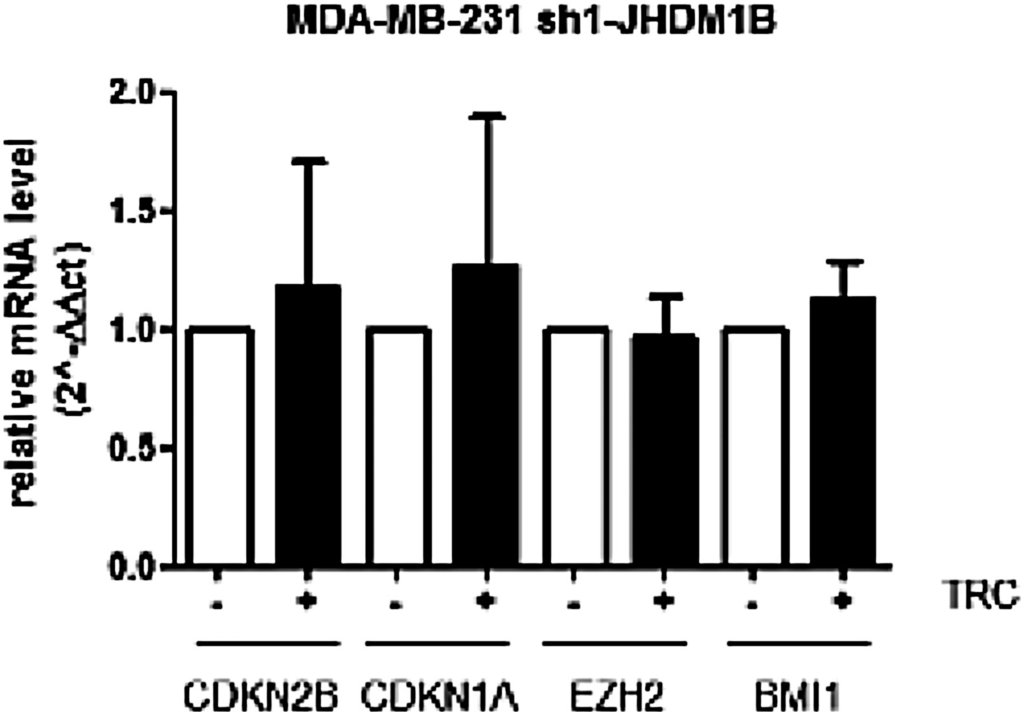

Supplementary Figure 1: Evaluation of genes expression in JHDM1B KD cells. JHDM1B KD in MDA-MB-231 sh1-JHDM1B does not cause a different gene transcription of CDKN2B (p15) and CDKN1A (P21) and genes implicated in the policomb silencing complex PRC (EZH2 and BMI1). Data were obtained by RT-PCR analysis after 6 days of TRC treactement (black filled) or in control condition (white filled) and statistical analysis performed by paired Student's *T*-test.

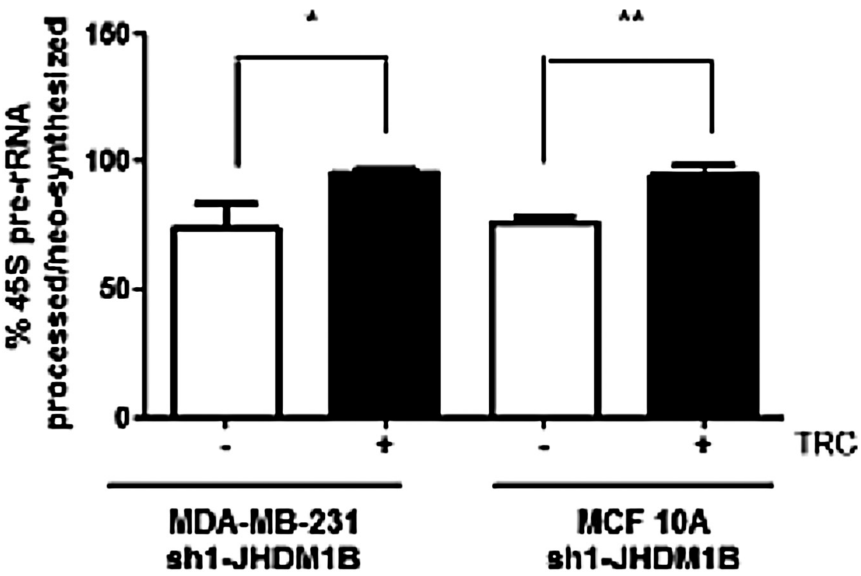

Supplementary Figure 2: JHDM1B KD improves 45S pre-rRNA processing. MDA-MB-231 sh1-JHDM1B control cells processed the 74,33 ± 16,5% of the total neo-synthesized 45S pre-rRNA against the 95,75 ± 2,78% in MDA-MB-231 sh1-JHDM1B KD; similarly MCF 10A sh1-JHDM1B processed the 76,6 ± 4,04% in control cells and the 94,95 ± 7,3% in KD cells . Data were analyzed by unpaired Student's *T*-test : *$P < 0.05$; **$P < 0.01$, ($N = 6$, error bars, SEM).

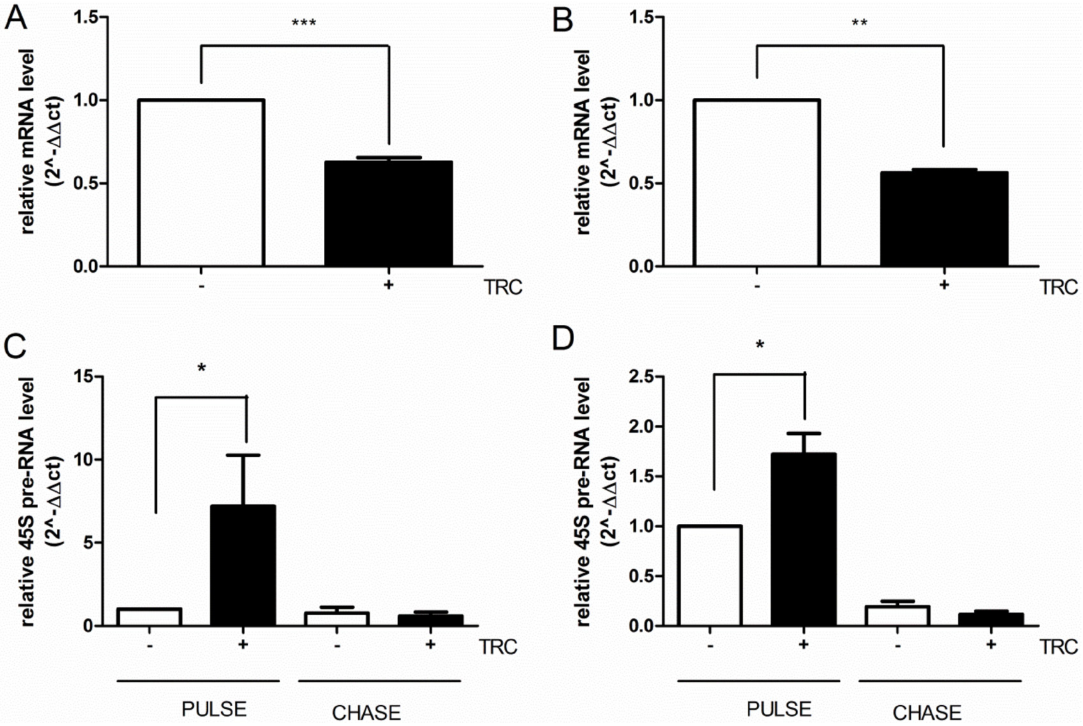

Supplementary Figure 3: Data validation in cells expressing a different shRNA against JHDM1B (sh2-JHDM1B): (**A** and **B**) Levels of JHDM1B mRNA after 3 days of TRC administration were assessed by real time RT-PCR: MDA-MB-231 sh2-JHDM1B (A) and MCF 10A sh2-JHDM1B (B). (**C** and **D)** Effect of JHDM1B KD on neo-sythetised 45S pre-rRNA production in MDA-MB-231 sh2-JHDM1B (C) and in MCF 10A sh2-JHDM1B (D). Data analysis performed with Student's *T*-test paired: *$P < 0,05$; **$P < 0.01$; ***$P < 0.001$, (error bars, SEM).

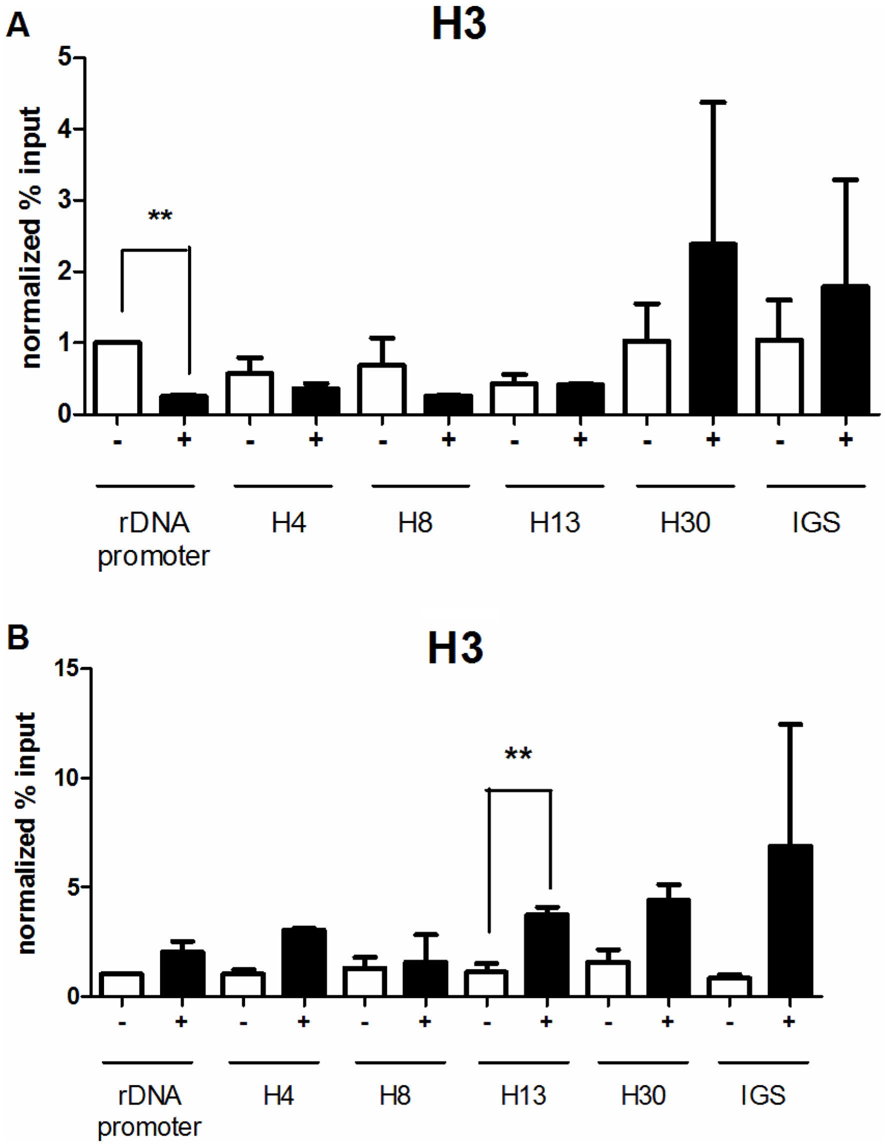

Supplementary Figure 4: JHDM1B knock-down cause a remodulation of total histone H3 association at the rDNA level. (**A**) Chromatin immunoprecipitation using specific anti-bodies against H3 histone in MDA-MB-231 sh1-JHDM1B control (white) and KD cells (black). (**B**) Chromatin immunoprecipitation using specific anti-bodies against H3 histone in MCF10A sh1-JHDM1B control (white) and KD cells (black). Quantifications were obtained by real time PCR with primers specific for different regions of rDNA. Data were expressed as % of the input normalized against the % of the input obtained for the rDNA region of control cells. The statistical analysis was performed with the Student's *T*-test pared $*P < 0.05$; $**P < 0.01$ ($N = 4$, error bars, SEM).

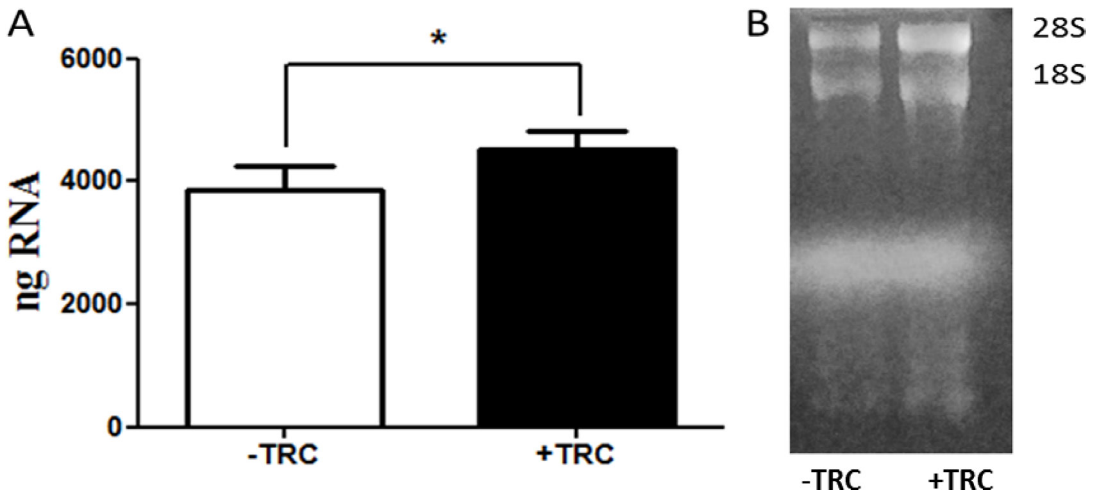

Supplementary Figure 5: (**A**). Average total RNA obtained from the same number of MCF10A sh1-JHDM1B cells (3*105 cells) treated (black filled) or not (withe filled) with TRC. Data analysis was performed with Student's *T*-test paired: $*P < 0.05$. (**B**) Denaturating formaldehyde 1% agarose gel loaded with the same volume of total RNA extracted from 3*105 cells MCF10A sh1-JHDM1B. Before loading the gel the RNAs were resuspended in the same final volume.

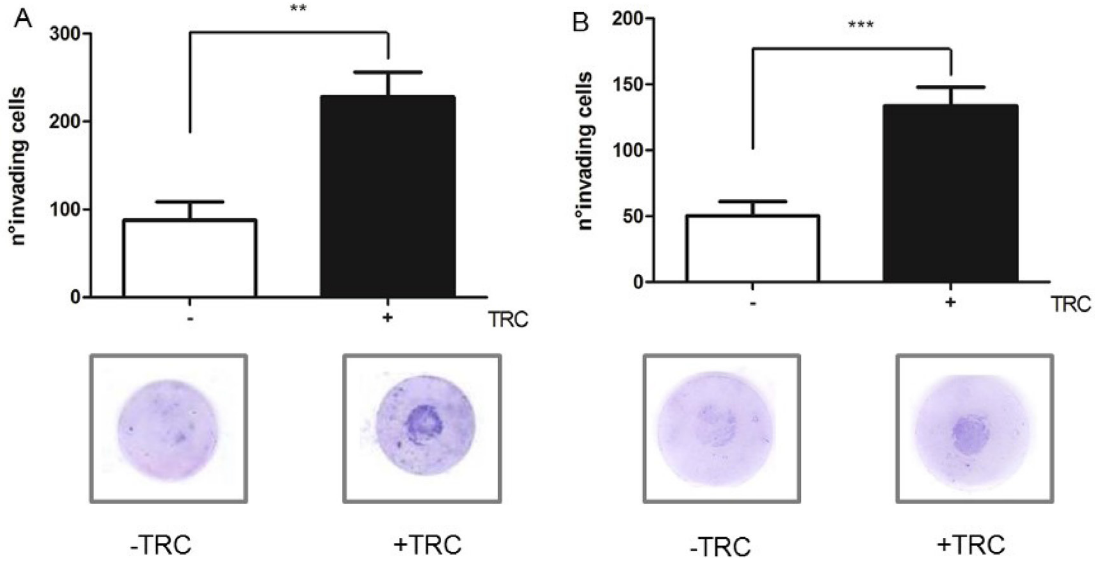

Supplementary Figure 6: Matrigel invasion assays in cells expressing a different shRNA (sh2-JHDM1B). (**A**) MDA-MB-231 sh2-JHDM1B control (white) and KD cells (black). (**B**) MCF 10A sh2-JHDM1B control (white) and KD cells (black). Photographs of 5 different areas were acquired for each filters and used for cell counts. Data analysis was performed with Student's *T*-test unpaired: $**P < 0.01$; $***P < 0.001$ ($N = 5$, error bars, SEM). Images below are representative of the matrigel coated filters after 16 h of invasion.

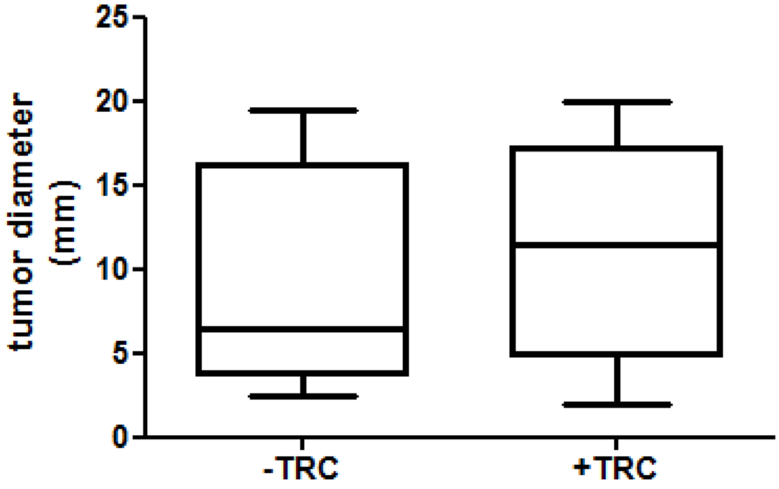

Supplementary Figure 7: Average tumors diameter in MDA-MB-231 sh1-JHDM1B Xenografts. MDA-MB-231 sh1-JHDM1B control (−TRC) and KD cells (+TRC) were injected on both flanks of 5 weeks old females Balb/COlaHsd-Foxn1nu. After additional 9 weeks animals have been euthanized, and tumor isolated and measured by a caliber, revealing a general incremental trend in tumor growth of KD Xenografts, but statistically un-significant chances in tumor diameter have been observed. Statistical analysis was performed by Student's *T*-test unpaired (*N* = 10, min to max).

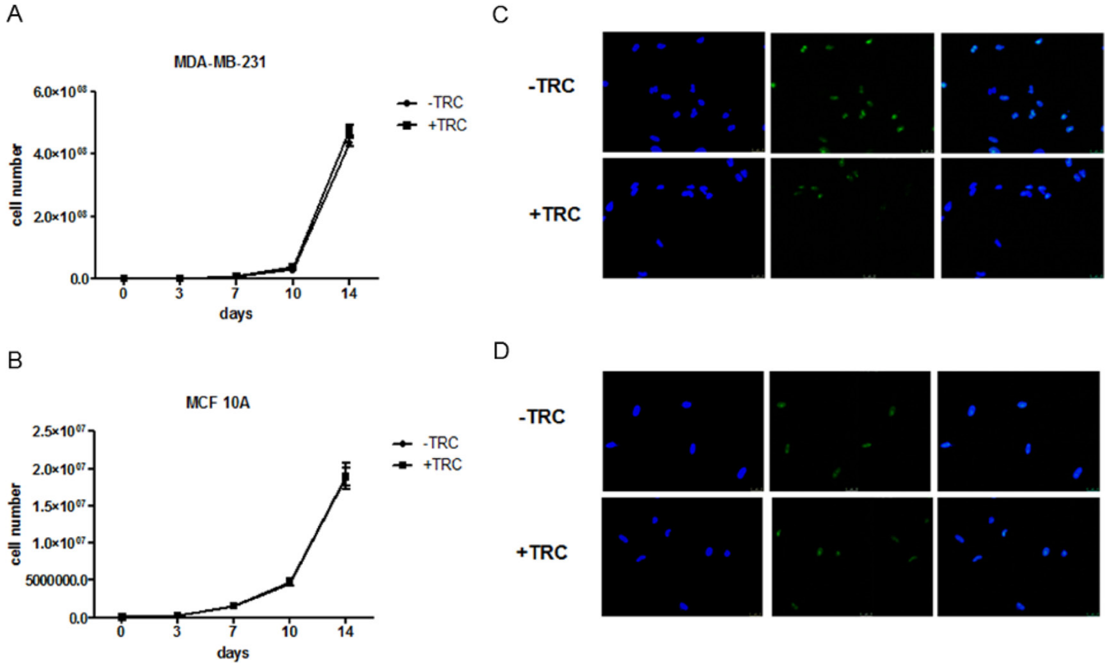

Supplementary Figure 8: Cell proliferation and 5-fluoro uridine incorporation of the wild type cell lines starting from 6 days of continuous 1 µg/ml TRC administration. (**A**) MDA-MB-231 cells growth profile of control cells (continuous line) and TRC treated cells (dotted line) revealed that TRC daily administration does not impact MDA-MB-231 cell proliferation. (**B**) MCF 10A cells growth profile of control cells (continuous line) and TRC treated cells (dotted line). (**C**) 5-fluoro uridine incorporation in nascent RNA of MDA-MB-231 treated or not with TRC. Cells were cultured with 5-fluoro uridine 2 mM in culture medium for 20 min and stained with a specific monoclonal antibody for halogenated uridine. After incubation with the anti-mouse Alexa 488, RNA synthesis can be observed looking to the green signal that localize preferentially at nucleolar level. No differences in rRNA synthesis has been found after 6 days of TRC administration. (**D**) 5-fluoro uridine incorporation in nascent RNA of MCF 10A treated or not with TRC, revealed no difference in rRNA synthesis between control and TRC treated cells. The graphs represent a technical replicate in which the levels of JHDM1B messenger measured by qPCR were 98% and 110% of the control, respectively for the MDA-MB-231 and for the MCF10A.
